# Anal metastasis of sigmoid colon cancer

**DOI:** 10.1002/ccr3.5909

**Published:** 2022-06-02

**Authors:** Takayuki Yamada

**Affiliations:** ^1^ Asunaro Clinic Takasaki City Gunma Prefecture Japan

**Keywords:** anal metastasis, computed tomography, diagnosis, excision, sigmoid colon cancer

## Abstract

A 73‐year‐old man underwent a stage‐Ⅱ sigmoid colon cancer resection. He was carefully monitored, and metastasis or anastomotic recurrence was noted 51 months later. The anal examination revealed a firm pink anal tumor, histologically identified as a metastasis of the colon adenocarcinoma.

## Graphical abstract

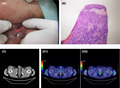

## 1 INTRODUCTION

Sigmoid colon cancer sporadically metastasizes to regional lymph nodes, liver, and lungs.^1^ However, intestinal metastasis secondary to colon cancer rarely occurs.^2^ Therefore, we did not anticipate this rare metastasis in the follow‐up period after surgery. The diagnosis of anal metastasis secondary to sigmoid colon cancer was not initially suspected in this case. The postoperative monitoring for surgical site recurrence and distant metastasis revealed no abnormal findings for 51 months. The anal metastasis was confirmed via digital rectal examination.

## 2 CASE HISTORY/EXAMINATION

**FIGURE 1:**
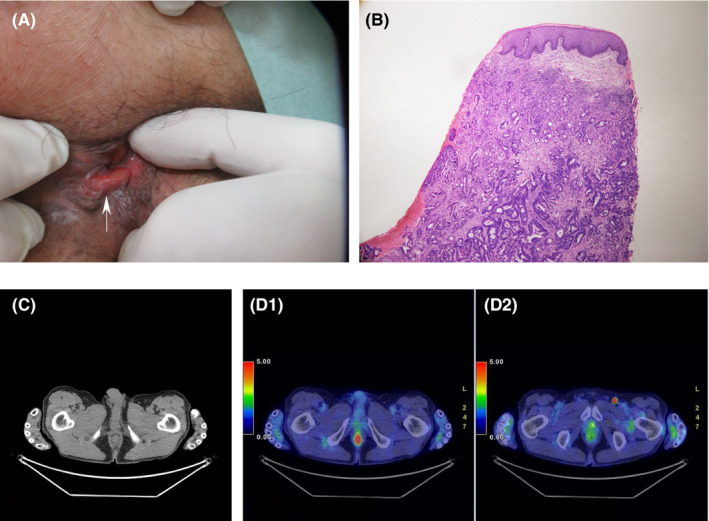
(A) An anal examination reveals a pink, firm anal tumor. (B) Biopsy sample shows the heterotypic columnar epithelium arranged in irregular duct‐like configurations, amalgamation duct‐like configurations, and comb‐like configurations under the stratified squamous epithelium. (C) Conventional CT did not reveal an anal tumor. (D) A positron emission tomography scan reveals an anal tumor (D1) and an inoperable left inguinal lymph node metastasis (D2)

A 73‐year‐old man underwent sigmoidectomy with lymph node dissection for stage‐Ⅱ sigmoid colon cancer (moderately differentiated adenocarcinoma, cT3cN0cM0 Stage IIa, pT3pN0M0 p Stage IIa, negative proximal, distal, circumferential, and mesenteric margins) four years ago. He was carefully monitored using chest and abdominal computed tomography (CT) scans semi‐anually, serum tumor markers at three‐month intervals, and colonoscopies once per year. Colonosocopic examination did not detect the anal tumor at the point of preceding diagnosis of anal metastasis five months ago. No evidence of metastasis or anastomotic recurrence was observed until four years and three months postoperatively, when he had an episode of hematochezia, initially associated with internal hemorrhoids. Anal examination revealed a firm pink anal tumor (Figure 1A). The biopsy showed heterotypic columnar epithelium, arranged in irregular duct‐like, amalgamation duct‐like, and comb‐like configurations under the stratified squamous epithelium. These findings suggested a metastatic tumor secondary to colonic adenocarcinoma (Figure 1B). The patient was treated with inhaled tiotropium bromide hydrate/olodaterol hydrochloride and oral theophylline. He became oxygen‐dependent due to pulmonary fibrosis and emphysema. Moreover, he had been taking oral prednisolone due to a history of rheumatoid arthritis, as well as oral diltiazem due to a history of an angina variant. Thus, he had significantly decreased pulmonary function, and rectal amputation was contraindicated. The complications of local tumor resection include a defective anus, loss of anal sphincter function, incontinence, and bleeding tendencies. Thus, the procedure was not performed.

## 3 DIFFERENTIAL DIAGNOSIS, INVESTIGATIONS, AND TREATMENT

The differential diagnoses for an anal submucosal tumor included carcinoma, lipoma, myoma, and lymphangioma. A biopsy was performed to confirm the diagnosis. Conventional CT did not detect the anal tumor at the point of diagnosis of anal metastasis six months ago. Additionally, colonosocopic examination did not detect the anal tumor at the point of the preceding diagnosis of anal metastasis five months ago. Conventional CT did not reveal an anal tumor (Figure 1C) and a left inguinal enlarged lymph node. However, PET‐CT images revealed an inoperable left inguinal lymph node metastasis and a submucosal anal tumor (Figure 1D1,D2).

Indeed, primary anal cancer is another differential diagnosis. However, this tumor mucosa was intact macrosopically, and adenocarcinoma cells were localized under the stratified squamous epithelium microscopically, indicating this tumor as metastatic. PET‐CT detected no abnormal accumulation in the lungs, pancreas, or gallbladder. The upper gastrointestinal endoscopic examination was unremarkable. The patient was presumptively diagnosed with anal metastasis secondary to sigmoid colon cancer. Magnetic resonance imaging was not performed because surgery was contraindicated for this patient.

## 4 OUTCOME AND FOLLOW‐UP

Since the patient's clinical status precluded surgical intervention, he received chemoradiotherapy for local control of the bleeding tumor. The bleeding has been well controlled, and the patient has survived the disease for eight months without anal tumor progression and is still alive.

## 5 DISCUSSION

Postoperative monitoring for recurrent cancers of the alimentary tract involves conducting chest‐abdominal‐pelvic CTs to assess for distant metastasis (lungs, liver, adrenal glands, bones, or spleen) or dissemination (ascites, pleural effusion, paraaortic or regional lymphadenopathy) yearly for four years. However, the conventional CT cannot detect lymph node metastasis, measuring <10 mm. Moreover, there are currently no objective radiologic criteria for lymph node metastasis.^3^ It is difficult to detect pinpoint, nodular, and circumintestinal involvement on CT. Furthermore, intestinal metastasis rarely occurs.^2^ The mechanism behind colorectal metastasis secondary to colorectal cancer has not been fully elucidated. Intestinal metastases, derived from submucosal vessels, typically present as submucosal tumors because the cancers are mainly located in the submucosa and the muscularis propria. In this case, a direct incisional biopsy was performed. In cases wherein a manual incisional biopsy is not feasible because of the tumor location, an endoscopic ultrasound‐guided fine‐needle aspiration or boring biopsy is indicated.^4^ These procedures require a specialized institution. It is difficult to detect this type of recurrence through conventional colonoscopic examination. It is important to routinely perform a digital rectal examination to monitor for postoperative colon cancer recurrence. Anastomotic recurrences have occasionally been reported, but this was not observed in the patient. Lymph node metastasis secondary to sigmoid colon cancer developed from the paracolic lymph nodes to the inferior mesenteric lymph nodes and paraaortic lymph nodes. It rarely metastasizes to the pararectal lymph nodes. This suggested that the tumor metastasized through the hematogenous route. During sigmoidectomy, his serum tumor marker including CEA and CA19‐9 were within normal levels. Therefore, in this case, serum tumor marker did not provide a benchmark of tumor recurrence.

He has lived 8 months after local chemoradiation therapy. His life prognosis remains unknown because this type of recurrence rarely occurs; however, tumor volume reduction before chemoradiation therapy or lymph node radiation may improve his life expectancy.

Given the limitations of his clinical status, the patient received chemoradiotherapy only for local control of the tumor bleeding. In 38 previous cases of anal metastasis secondary to colorectal cancer, none were treated with chemoradiation,^5^ and the life expectancy for the patients remains unknown. In this patient, who presented with inoperative lymph node metastasis, palliative chemoradiation is a reasonable treatment option. The patient in this case was presumptively diagnosed with anal metastasis secondary to sigmoid colon cancer.

Anal metastasis secondary to colon cancer rarely occurs. This report guides clinicians to identify this rare type of metastasis.

## AUTHOR CONTRIBUTIONS

Takayuki Yamada: first author and diagnostician.

## CONFLICT OF INTEREST

None.

## ETHICAL APPROVAL

Ethical approval was not required for this study.

## CONSENT

Written informed consent was obtained from the patient to publish this report in accordance with the journal's patient consent policy.
